# Case Report: “*I got my brain back*” A patient’s experience with music-induced analgesia for chronic pain

**DOI:** 10.3389/fpsyg.2023.1141829

**Published:** 2023-04-28

**Authors:** Roberto E. Mercadillo, Eduardo A. Garza-Villarreal

**Affiliations:** ^1^Universidad Autónoma Metropolitana, Iztapalapa, Mexico City, Mexico; ^2^CONACYT, Mexico City, Mexico; ^3^Instituto de Neurobiología, Universidad Nacional Autónoma de México Campus Juriquilla, Queretaro, Mexico

**Keywords:** music, pain, opioids, clinical, music-induced analgesia, phenomenology, therapeutic accompaniment

## Abstract

Listening to music has progressively been proposed as a complementary alternative for chronic pain; understanding its properties and its neurobiological bases is urgent. We show a phenomenological investigation of a woman who has lived 20  years with chronic pain. The inquiry involved her experience of the context in which she listens to music, the intensity and quality of pain, body mapping, memories, emotions, and cognition. The participant listens to music for different reasons, such as pain and anxiety relief, motivation to exercise, and quality of sleep, but all seem to revolve around different strategies for pain management. Experiences in physiological and cognitive aspects included perceived restorative sleep that may have improved the participant’s general wellbeing and improved cognitive and motor performance as well as communication skills. The music enabled the participant not only to relieve pain but also withdrawal effects after discontinuing her opioid-based treatment. These effects may encompass endogenous opioid and dopamine mechanisms involving natural analgesia associated with pleasurable experiences. Future studies could consider phenomenological case studies and therapeutic accompaniment to reorient subjective properties of pain and expand quantitative and qualitative knowledge for more comprehensive reports on music and analgesia.

## 1. Introduction

Even with proper therapy, a high number of patients with chronic pain experience negative consequences that affect their health, self-perception, interpersonal relationships, and life in general (Veehof et al., 1999). In this context, music listening has been progressively proposed as an add-on alternative due to its positive effects on pain, anxiety and depression symptoms, and its easy access. Understanding the analgesic effects and its neurobiological basis is urgent, but it still faces difficulties concerning experimental designs, especially to model placebo effects (Rosenkjær et al., 2022). Nevertheless, several studies have confirmed that listening to music reduces acute and chronic pain (see Garza-Villarreal et al., 2017; Lunde et al., 2019 for a review), hence the name “music-induced analgesia.” For example, patients with low back pain showed improvement using music therapy (Guétin et al., 2005), and there is evidence of post-operatory analgesia using music (Kavak Akelma et al., 2020). A more recent study in mice showed that sound can induce analgesia via an auditory cortex–thalamus circuit (Zhou et al., 2022); however, there seem to be other mechanisms at play. The music chosen by the patients themselves (Li et al., 2011), valued as pleasant (Roy et al., 2008), and familiar (Shih-Tzu et al., 2010; van den Bosch et al., 2013), seems to be the most suitable for producing analgesic effects. However, the musical choice depends on multiple factors involving the individual history and culture. Its effects comprise imagination and metaphors that can only be understood subjectively. In addition, there are a few spaces where people living with pain or who have tried alternative therapies can share their experiences without stigma (Miglio and Stanier, 2022).

We show a phenomenological inquiry of a woman who has lived 20 years with chronic pain. After suspending her opioid-based treatment, she found music as an alternative for relief. Her experience is discussed considering neurobiological knowledge about pain and music. Therapeutic accompaniment is proposed as an alternative to understand similar experiences and to incorporate them into clinical and scientific fields.

## 2. Case description

The participant was a 58-year-old woman, a single mother of two adult children, and a grandmother of five grandchildren. She has a degree in accounting and a degree in environmental sciences. She was educated in music; she began to play the piano and the flute as a child and learned the guitar and saxophone in a self-taught way. She now works as a tax field auditor in the United States. She has been a chronic pain patient and has spent about 20 years on high doses of narcotic pain relievers after a car accident that caused her lower spine problems and created chronic pain.

In September 2021, she stopped taking medication “cold turkey.” She found that music not only helped with the pain, but she also believes it repaired her “brain chemistry,” which she felt to be affected due to long-term use of high doses of opioids, as well as the withdrawal symptoms.

Sharing her experience at the pain clinic, she was told initially that music was just a distraction and subsequently that she was very creative in using music to relieve pain, but she did not agree with that statement at all and was convinced that music works differently than only distraction. After observing the insistence of the participant on the effects of music to relieve pain, her physician from the Comprehensive Pain Program provided her with a research article reporting that music reduces pain and increases functional mobility in fibromyalgia (Garza-Villarreal et al., 2014). She was very intrigued about it, so she contacted the authors to share her experience and understand how music helps her manage chronic pain and gives her energy and motivation.

The study presented here was designed following the guidelines of the American Psychological Association (2002) and the Declaration of Helsinki and was approved by the Bioethics Committee of the Institute of Neurobiology of the Universidad Nacional Autónoma de México.

## 3. Diagnostic assessment

After having contacted the participant and knowing her case, a first open and free interview was carried out. In it, she shared her experience about her accident, the chronic pain she suffered, her diagnosis, treatment, and cessation of it, as well as the way she accessed music to deal with pain and her doubts about scientific research in this regard. A first analysis of this interview made it possible to identify the most relevant points for the participant and to develop two guides to investigate her experience of pain over 20 years in two conditions: without medication/with medication or with music. The aspects evaluated in both guides were as follows: intensity and quality of pain; body mapping; memories associated with pain; emotions; and cognition (perception, attention, learning, memory, and language). Each of the guides was answered in writing by the participant with the support and advice of the researcher. Subsequently, each of the points addressed in the guides were expanded verbally and developed in two interviews.

Then, the participant was instructed to make a list of the musical pieces that she considers most representative to relieve her pain and a list of the representative musical pieces that caused her pain. For each of the musical pieces, the participant indicated: the context in which she listens to that music; memories (if any) evoked; appeared images (visual, tactile, or auditory); emotions or feelings; parts of the body—in addition to the relief of the lower back and hips—that show sensations or experiences when listening (the way that piece of music makes her body feel). The participant indicated these elements in writing. In subsequent interviews, each piece of music was jointly listened by the participant and by the researcher, and each of the indicated aspects was expanded verbally and developed through questions directed by the researcher.

The complete investigation was carried out for 3 months. All interviews were conducted via Zoom and were recorded for later analysis covering a total of 6 h. Each experience guide and each list of music that relieves and causes pain was answered in 3 weeks.

Next, a third-person narrative and some testimonials respecting the explanatory style of the participant herself are present to understand, from her perspective, what she considers the most relevant from her experience. The narrative follows the neurophenomenological proposal that makes use of the experience communicated in first-person testimonials and emphasizes embodiment as a substrate of individuality (Varela et al., 1991; Díaz, 2022). We encourage the reader to review the more extensive narrative with verbatim illustrative testimonials shown in the Supplementary material.

### 3.1. Accident, treatment, and “cold turkey”

In 2001, a large car coming down a hill lost control and crashed into her, and pushed her car onto a busy street. At first, it did not hurt, but 6 h later she had excruciating pain in her spine and could not walk and then she began to feel sharp like shards of blast sticking. She received two sets of facet joint injections. When she stated that the second set of facet joint injections increased the pain in her lower spine and hips she was told, “*it was a steroidal flare but no doctor can currently tell me what it was.*” The pain increased almost permanently, and that is when she got her first prescription for oxycodone.

Her pain was mostly in the lower spine, and it felt like a type of crushed glass or like there was an ice pick in the middle between L3 and L4. She has a herniated bulging disk, nerve compression stenosis, and arthritis. Her second and third toes on her left foot have been tingling for 20 years since the accident and about 5 or 6 years after she developed sciatica.

She participated in a Comprehensive Chronic Pain program for the first time in 2019, when she was taking high doses of OxyContin. There they offered multidisciplinary rehabilitation, reiki, massages, and acupuncture, but she found no relief and she was angry about not finding alternatives to medication. As of September 2021, she was taking nearly 300 mg of extended-release OxyContin and 300 mg of immediate-release oxycodone so, she went cold turkey and that was the last time she took oxycodone. Intuitively, she thought she needed music with headphones and as the music played she felt like it was repairing or feeding her brain. She is currently using music, reiki, acupuncture, and Buddhist meditation for pain.

### 3.2. Pain experience, emotions, and cognition

**Figure 1:**
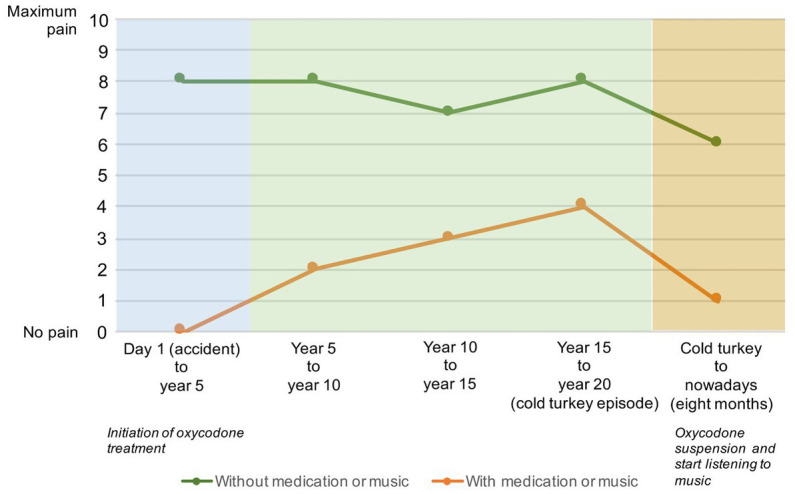
Pain intensity over 20  years from the accident to 8  months after the cold turkey episode: green line—intensity when the participant used medication (oxycodone from the 8th month after the accident to year 20 just before the cold turkey episode) or listened to music (at the end of year 20 to 8th month after at the moment of the assessment). Orange line—intensity when the participant did not use medication (before the oxycodone treatment and some rarely days when she suspended the treatment for a long 20 years) or did not listen to music to relieve the pain. At first, 8 months before the first dose of oxycodone, she felt an intensity of 8 and then fluctuated to 7 when she began to be more active, riding a bike, and having a natural path by eating anti-inflammatory foods and avoiding sugar and salt. When she started using the medication, she felt no pain at first, but it increased right before the cold turkey episode. Since she listens to music, she has had periods completely free of pain, sometimes 1 or 2 days without pain, but with an intensity 6 if she does not listen to music: “*if you ask me where my pain average was in the last 2 months I would say maybe a 3 in the worst period… Once in a while, if I go hiking up high elevations may be 6 or 7. But every day at my desk call week is 4 maybe.*”

**Figure 2:**
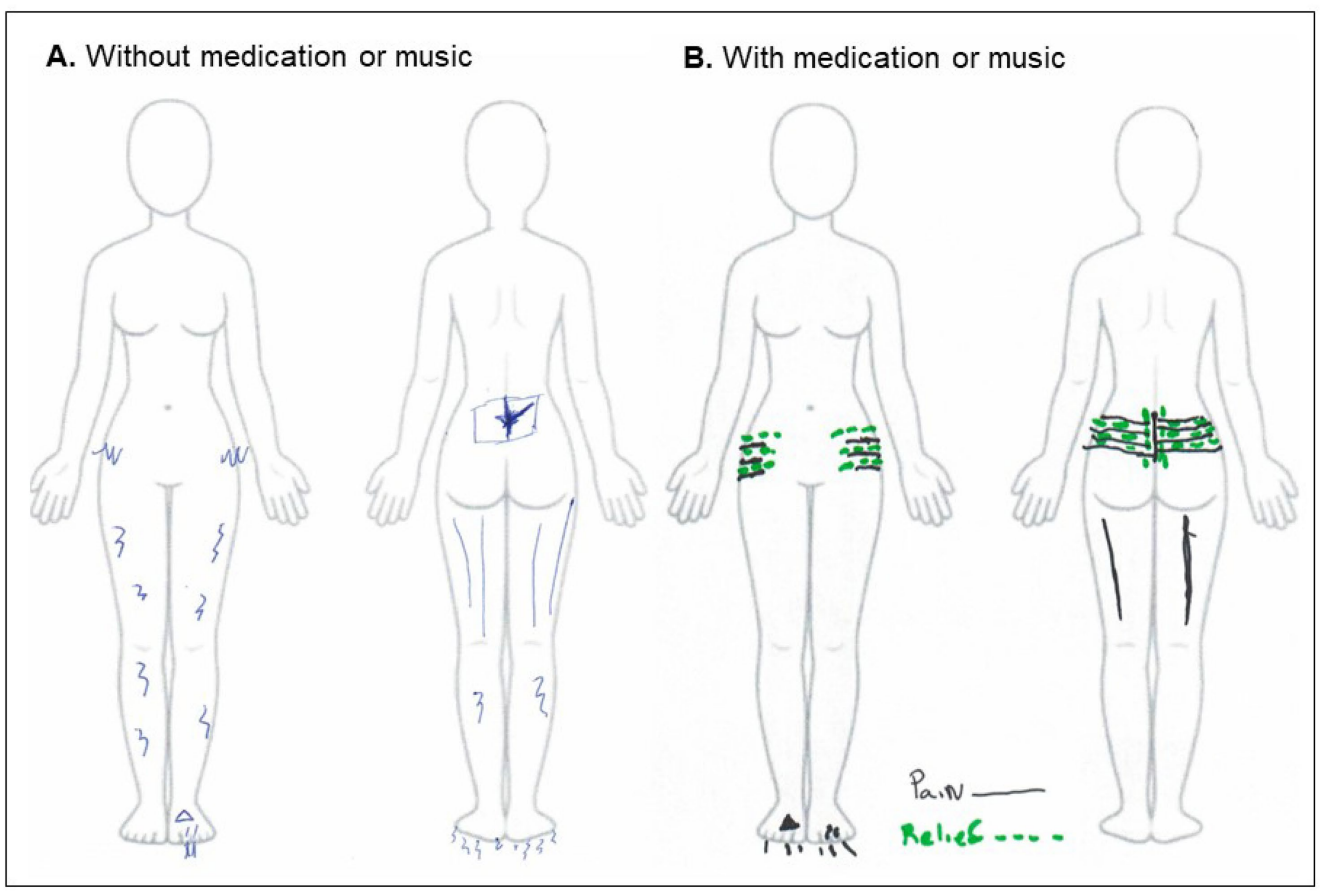
Location of pain when she has not used medication **(A)** and when she took medication or listened to music **(B)**. When there were no periods of medication, the pain was felt in the back of the legs, which was sciatica, and nerve pain in the legs and under the feet. When she took medication or listened to music, she felt pain in her lower spine and feet, where she also felt relief.

Pain intensity and body location have fluctuated throughout these 20 years of treatment (see Figures 1, 2).

She has experienced a variety of emotions throughout these 20 years, mostly negative: exhaustion, stress, sadness, anger, frustration, and impatience.

She has also felt happiness and relaxation with pain relief, either from medication or music, but even when happiness was present, other emotions persisted when using medication: stress, isolation, anger, and frustration with the pain. Sleep was a prominent issue since she had insomnia for a long time, but now with music sleep has improved.

Before the music, her sense of touch was dulled, whether she felt pain or relief. With the music “*not only* [her] *sense of touch came back but the gray could went away.*”

She had difficulties concentrating while using narcotic medication. Using music “*has given* [her] *much more energy to pay attention to the things that matters…*.”

In terms of learning and memory, she used to have a hard time remembering things and focusing on new material at work. With music, she has increased her learning ability and is alert with an overdose of joy and interested in everything again.

### 3.3. Music that relieves and causes pain

Now, she probably uses music almost every waking hour, except when she is in a meeting. She has approximately 80 playlists, and each playlist can have 15–100 tracks. From there, she can make lists of music to relieve pain but also to know what music causes her pain. She uses the tidal streaming service because they have a large library of music. However, the 3D music most streaming services have sounds like the band is playing in a gym with bad acoustics. High-fidelity headphones are crucial for good sound quality and the noise cancelation enhances the sound quality, so she can only hear the music. Although she has a stereo and albums, the stereo or the CD player is not effective.

Every day she tries to find music that relieves the pain, so she is always exploring what kind of rhythms, tones, and pitches are helping. In addition to sound properties, her exploration involves memories and physical sensations, and moods evoked by the songs, as well as certain body positions. With this, she can decide the most appropriate times to listen to each song. The experiences associated with each of the five songs that alleviate her pain the most are shown in Table 1 (more extensive testimonials in the Supplementary material).

**Table 1:** List of musical pieces that relieve the participant of pain the most, the context in which she listens to them, and associated experiences.

Music that relieves pain						
“Piece” Musician or band	Context in which the piece is listened	Memories (if any) this piece brings to mind	Visuals this piece brings to mind	Feelings this piece causes	Part of the body in which this piece is perceived (in addition to relieving pain in the lower spine and hips)	How this piece makes the body feel
“Golden” Alexis French	“When pain is the worst and I have to lay in a fetal position. I am usually lying in a fetal position on the bed”	“No memories. This is new music”	“I visualize gliding/flying among the clouds above an ocean shoreline”	Joy, peace, freedom, beauty.	“I feel this song throughout my entire body”	“It makes my body feel like it is floating and gently rolling on waves of clouds. My body feels light and in my mind it feels like the keys are tickling my entire body”
“Confines” (official live studio session with string quartet) Black Pumas	“At my desk, out running errands or laying in fetal position when pain is the worst”	“No memories. This is new music. My mind is absorbed in the notes and layers of notes”	“I visualize the instruments being played and the notes on sheets of music and just grooving to the beat”	“The string quartet and Eric Burton’s singing voice is soothing and relaxing. The beat is also soothing and relaxing”	“The beat, the string quartet and Eric Burton’s singing release pressure from my lower spine and relaxes my entire body. My body feels like it absorbs the music and is being uplifted and gently rocked to the beat”	Relaxed and soothed
“Know you better” (official audio version) Black Pumas	“At my desk or laying in fetal position when pain is the worst”	“No memories. This is new music. My mind is focused on the ticking of the percussion. However, it does remind me of when I would go to the underground dance scene in Chicago where Frankie Knuckles created Chicago house music. His live sets could put an entire crowd of 500 people into a trance. These were similar to raves, but before raves and better music (no electronic or techno crappy music)”	“I visualize the ticking of the percussion…a drum stick ticking against the rim of a snare drum and the other instruments being played”	“The ticking of the percussion focuses my mind while the singing and beat of the other instruments relieves stress as well as the pressure in my lower spine”	“I feel this song mostly in my mind in addition to relieving pain in my spine. It is very useful for increased pain that is caused by stress”	Relaxed
“The Panther” Manu Dibango	“I listen to this song when I am running either at the gym or outside and I start to feel increased pain”	No memories	“I visualize myself running faster and farther down a road”	Energetic	“I feel this song throughout my entire body”	“This song makes me feel stronger and more energetic and helps me to run through the pain”
“Ave Maria” Schubert Ave Maria with Renee Fleming	“This song is particularly helpful for increased pain from stress such as when stressed out in crowded stores, waiting in long lines or stress at work. I also use this song when I have to lie in a fetal position when pain is the worst for any reason -either increased pain from mechanical issues or stress”	“Visiting the Sistine Chapel and the Colosseum in the Vatican City and Rome”	“The Sistine Chapel, high, vaulted cathedral ceilings, the interior of the church I attended as a child; it was a beautiful church but the memories are not so great so I do not think of those”	“The sensation of being connected to the universe and the energy of all things, past, present and future”	“I can feel the singing along my vertebrae and hip bones gently pulling the pain out”	“Soothed, and releases pressure in my lower spine. It also makes my body feel like it is floating”
“The flower duet” Charlotte Church	“This song is also particularly helpful for increased pain from stress such as when stressed out in crowded stores, waiting in long lines or stress at work. I also use this song when I have to lie in a fetal position when pain is the worst for any reason -either increased pain from mechanical issues or stress”	No memories	“No visuals really, my mind is absorbed in the music”	Peace, calmness, optimism, and hope	“I feel it rolling throughout my inner body”	Weightless, gliding, and soothed
“Maybe tomorrow” Stereophonics	“This song works best for pain due to stress”	“Hitchhiking across the United States, being on the open road, traveling”	“Driving down an open road along the ocean coastline in a convertible, or being on the open road”	Freedom, happiness, serenity, optimism	Chest/Heart	“Releases stress and tension in my entire body. The vocals and the beat are soothing to me”

Her exploration has also allowed her to identify music that exacerbates her pain and that reminds her of social situations evoking anger or stress. So, she has built a list of music she avoids, as shown in Table 2 (more extensive testimonials in the Supplementary material).

**Table 2:** List of musical pieces that most cause pain to the participant and associated experiences.

Music that relieves pain						
Piece musician or band	Context in which the piece is listened	Memories (if any) this piece brings to mind	Visuals this piece brings to mind	Feelings this piece causes	Part of the body in which this piece is causes pain (in addition to lower spine and hips)	How this piece makes the body feel
“The Bigger Picture” Lil Baby	“Do not listen to this song as it causes me pain”	“This song reminds me of when my daughter was brutalized by a South Burlington, Vermont police officer for what is called in the Black community—“The crime of driving while Black” or more commonly known as “DWB”. It also reminds me of how every time my son is out driving whether he will make it home safely. Having to give your children talks on how to remain safe around police because they are Black runs deep in how you see certain aspects of this society and is stressful. It also reminds me of how the University of Vermont police would pull us over if my son was in my car just to run our IDs”	“The day my daughter was brutalized by a South Burlington cop and how I had to be pulled out of the hospital trying to stop them from doing more harm at the request of the officer” “Getting pulled over by the Jersey police every time we took the Jersey Turnpike to visit my friend’s mother in New York City. It got so bad that if he was driving I would lay down in the seat so I could not be seen while on the Turnpike or he would if I was driving” “Seeing a Chicago cop beat a small child in the back of a police car”	Anger, Outrage, Profound sadness	“Primarily in my chest as well as lower spine”	“Tense and heavy pressure on my chest”
“Shelter” Vic Mensa, Wyclef Jean, Chance the Rapper	“Do not listen to this as it causes me pain”	Living in the projects in Chicago with my children where there were shootings every day and gang fights. Children had been shot and died right in front of my building. The police only came to our neighborhood to hunt and for target practice and no ambulance would come either” “Remembering how my daughter’s school bus went right through the Cabrini Green high rises where there were snipers on the rooftops and how when my neighbor stood up against the Latin Kings they came and shot him and his son”	“My old neighborhood in Chicago”	“Profound sadness and anger about the way things are”	“Throughout my body in addition to my lower spine”	Heaviness, Physical exhaustion, Pain
“Unaccompanied Cello Suite No 1 in G Major” Yo-Yo Ma	“Do not listen to this as it causes me pain”	No memories	“Nails down a chalkboard”	“The scratchiness of the strings makes my skin crawl”	“The back of my neck, jaw and all the way down my spine”	“Tightness, clenching of jaw, uncomfortable to my ears”
“Overture from the marriage of Figaro” Mozart	“Do not listen to this as it causes me pain”	No memories	No visuals	“The quick build ups, then crashing loudly and at other times the build up and left hanging of the instruments along with the overall aggressiveness of the piece is stressful, too aggressive and too busy. It also has too many short musical phrases that go nowhere other than to connect to another, but different, phrase, the discontinuity is disorienting and stressful”	“In addition to my lower spine, it gives me a headache. It also bothers my eyes a great deal. When I am listening to music while relaxing I usually close my eyes to focus solely on the music, this piece makes me feel like my eyes are rolling around in their sockets”	Nauseated
“Houses of the Holy” Led Zeppelin	“No longer listen to this particular song by Led Zeppelin as it causes pain”	“Keg parties in high school”	No visuals	“The slightly discordant repeated guitar riffs hurt my ears and cause tension to the point it makes me tighten up my shoulders. The high pitched somewhat screeching vocals later in the song do the same”	“It hurts my ears in addition to my lower spine”	Tension

## 4. Discussion

This case shows the experience of a person who found in music an alternative to alleviate her chronic pain. However, her first experience was not with pain but withdrawal effects after stopping opioid-based medication used for 20 years. Though medication provided her with pain relief, it also altered her perception of her body and emotions, making it difficult to experience wellbeing and perform daily activities. Although there is some evidence that music helps reduce substance abuse and craving, studies are not conclusive and its effects are not explained by the neurochemical mechanisms involved in the substances, but rather by positive feelings elicited by music that may decrease fear and guilt, and facilitate acceptance (Mays et al., 2008; Ghetti et al., 2022). A possible mechanism could involve endogenous opioids and dopamine released when listening to music and whose effects involve natural analgesia processes associated with pleasurable experiences (Lunde et al., 2019). However, a recent study showed that in acute pain, the expectation of analgesia predicted pain relief with music, even when blocking dopamine and endogenous opioids (Lunde et al., 2022). As for chronic pain, the mechanisms of music-induced analgesia are still unknown. Placebo effects could account for the music effect and have been previously suggested to be involved as part of the cognitive mechanisms of analgesia (Lunde et al., 2019). For example, we have shown that expectation of analgesia predicts levels of music-induced analgesia (Lunde et al., 2022), and expectation of analgesia is a known and complex mechanism involved in placebo effects (Rosenkjær et al., 2022).

Music-induced analgesia has been suggested to involve autonomic modulation through the descending pain modulatory system, elicited by top-down regulatory processes (Salimpoor et al., 2011; Garza-Villarreal et al., 2014, 2017). Such processes may contribute to positive emotional experiences associated with bodily sensations, as shown in phenomenological reports (Eschrich et al., 2008), as well as in the participant’s testimonials referring to a variety of relief sensations in her spine, legs, and feet according to each type of music listened at different times and contexts. Imagination on the body may constitute a top-down element so that pain or relief experiences become figurative providing awareness and control over them (Miglio and Stanier, 2022).

An interesting note is a frequency with which the participant uses music (almost every waking hour), which contrasts with sessions proposed every so often (e.g., 20–30 min. daily; Garza-Villarreal et al., 2017). Additionally, she indicates certain instrumental properties, such as the acoustic quality and the use of high-fidelity headphones to elicit analgesia. Such testimonials may advise about the required frequency and instruments to produce and maintain analgesic effects. The complexity of analgesia includes the neurobiology linked to listening to music. This involves not only the auditory system and cortex but also its projections toward cortical and subcortical regions, such as the orbitofrontal cortex or the amygdala, which allow acoustic analyses of pitches, tones, rhythms, intensity, and roughness, as well as feelings and contexts (Boso et al., 2006). Thus, analgesia may involve expectations, attention, contexts, and moods that define the relief experience (Bingel and Tracey, 2008). That is why knowing the history of the person is crucial to know musical effects. Our participant’s musical training feasibly influenced her ability to transfer auditory emotional experiences to other sensory modalities experiences (e.g., visual or tactile), as it is observed in trained musicians (Logeswaran and Bhattacharya, 2009). Moreover, it may have influenced her intuition to use music to relieve opioid withdrawal, to identify pieces with certain harmonies, pitches, tones, and rhythms that relieve pain, to appreciate music in a more emotionally and cognitively sophisticated way, and to avoid discordant pieces provoking her pain and evoking stressful situations (e.g., on social injustice).

In addition to positive feelings, improvements in physiological and cognitive aspects were mentioned. At this moment, the participant’s sleep is perceived as continuous and restorative. It has been suggested that certain music favors the synchronization of biological rhythms with the beat structures in music and this produces relaxation and allows attention to be focused on synchrony but not on the stressful situation (Dickson and Schubert, 2019). Sleep is essential for proper endocrine and immune function, cognitive restoration, and memory consolidation (Zarcone, 2000; Stepanski and Wyatt, 2003). Therefore, better sleep may have provided the participant with general wellbeing and improved cognitive and motor performance during the day. The music chosen by the participant may also influence this improvement since listening to music perceived as pleasant perhaps modulates long-term episodic memory and enables memory formation and retrieval (Eschrich et al., 2008). Perhaps, associative learning on non-painful experiences in everyday life is present.

An interesting effect was the improvement of communication skills. Socialization implies a rewarding dynamic in itself (Berridge and Kringelbach, 2008). Meanwhile, music is suggested to be closely related to social perception and reward (Savage et al., 2020). Social rewarding may contribute to building spaces in which the participant expresses her pain with less stigma (Miglio and Stanier, 2022) and reinforces her learning of new experiences. In addition, some alternative practices performed by the participant may contribute to increasing the effects of music. Though there are a few studies on Reiki and its mechanisms are unknown, it has been increasingly used for pain and anxiety relief (Billot et al., 2019). Some acupuncture techniques have been reported to induce analgesia and to reduce the use of opioids for pain relief (Akça and Sessler, 2002). Meditation induces alleviation in chronic pain patients and helps reduce cravings. It elicits neural activation associated with maintaining attention and controlling bottom–up processes (Lee et al., 2012), so that it may contribute to reducing pain-related psychological effects and sympathetic reactivity related to anxiety and depression. In addition, meditation improves sleep quality, increases positive effect, and increases parasympathetic activation through relaxation and the cultivation of metacognitive resources, such as acceptance, resulting in perceptual distance from painful and distressing sensory and psychological stimuli (Britton et al., 2010; Amutio et al., 2018).

Across the interviews, the patient stated the use of music for different aspects of their ailment: for pain and anxiety relief, for exercise motivation, and for sleep quality, yet they all seem to revolve around different strategies for pain management and overall wellbeing. The fact that she mentioned that meditation was not possible before the therapeutic use of music suggests that music could also be used as a first approach to other aspects of the pain treatment like exercise or meditation.

Our participant’s experience illustrates the complexity of pain that encompasses physiological, cognitive, and social spheres shaping the history and subjectivity of the person experiencing pain. The understanding of the effects of music to relieve pain and its neurobiological mechanisms must consider that history and subjectivity. For this, therapeutic accompaniment may be useful.

The accompanier constitutes a mediating figure between the patient and the institution. Originally proposed for psychiatric institutions with outpatient treatment, the accompanier performances as a health and psychosocial daily life monitor that allows the patient to reorient her/his subjectivity and to follow her/his experiences during the treatment. Moreover, it helps to communicate the experiences and systematize them for the better understanding of both, the patient, and the medical institution to design more sensitive treatments and research (Watkins, 2015; Rodríguez et al., 2019). In psychiatric patients, the accompaniment has revealed that music favors the reconstruction of identities, social integration, self-esteem, anxiety reduction, and motor performances (Andrade and Pedrão, 2005), as shown by our participant when expressing her experience in current physical activities. When reviewing her experience at the end of the interviews, the participant told the researchers her concern about what would happen if she stopped listening to music: *Would the effects on pain be lost? Would I feel pain all the time again?* There was no information in this regard to give the patient an accurate answer, but therapeutic accompaniment would help to reduce her anxiety in this regard and, perhaps, to gradually interrupt listening to music all the time to assess the durability of its effects. Therapeutic accompaniment is particularly relevant to the phenomenological method used in this study. The clinical instruments to assess pain expressions are limited and mostly made through scales and measures based on retrospective information. Subjective retrospective scales frequently coincide with other clinical or physiological measures, but as widely discussed by Miglio and Stanier (2022) on pain, and Copoeru (2014) on addiction, they displace the patient’s story. Thus, the qualitative dimension that phenomenology provides about the entire patient experience complements retrospective limitations by providing imagination and metaphors used by the patient to conceptualize, represent, and express pain. The phenomenological information can be supplemented by the patient’s clinical history to provide greater precision about their treatment experience over time.

In our opinion, the patient’s analgesia seems to stem from multiple causes and mechanisms. For her, music appears to be the first and the strongest form of analgesia in addition to medication. Without the side effects of opioid medication and maintained analgesia with the music, this seemed to have opened possibilities for the patient to engage in other activities that are known to reduce pain such as exercise (Lesnak and Sluka, 2020) and meditation. The feeling of self-control over her body and pain may have further increased her pain threshold (Tracey, 2010), and the reduction of depression and anxiety symptoms together increased her quality of life.

Abruptly stopping opioid medication is not advised due to the potentially unbearable opioid withdrawal symptoms ranging from 7 to 14 days, depending on the medication. Although research shows symptoms are not life-threatening, there is a risk of relapse and binging, which can be life-threatening (Pergolizzi et al., 2020). We suggest that patients wanting to try listening to music as an add-on treatment and reduce opioid medication ask their physician to down-titrate (slowly lower) medication to avoid or reduce withdrawal effects. We also suggest to remember that not everyone may benefit from music and more research is needed.

In conclusion, here we showed the case of a patient with chronic pain who reported to stop the use of opioid medication and changed to music listening as her main analgesic intervention, which allowed her to further engage in exercise, meditation, and other activities. Her case suggests there may be individuals with chronic pain who may greatly benefit from music-induced analgesia and that it may be possible to use music to reduce any type of pain medication. Cognitive and emotional mechanisms of analgesia seem to be present in our patients, with the implication of the descending pain modulatory pathway. Scientific studies of music-induced analgesia focus on average or group results; however, we believe that single case studies and interviews could help fill in gaps of knowledge that may not otherwise be possible with group studies. Ideally, future studies should consider both strategies as complementary and perhaps describe quantitative and qualitative results for a more integral report. Physicians should also study how music may reduce pain medication, with the future goal of a more integral treatment. There are still more questions than answers; however, with the help of patients like her, we may further understand chronic pain and music-induced analgesia.

## 5. Patient perspective

The participant provided her signed informed consent after the nature of the study was explained. She reviewed the draft of the manuscript to verify the proper comprehension of her communicated experience and to remove personal or institutional information she did not agree to present. The manuscript was submitted to be published just after her approval. In her own words: “*I have completed reviewing the article, it is wonderful! I wish there was a definitive mechanism for how music is working for me but I will remain overjoyed that it does in fact work for me and leave those mysteries of the mechanism to future research. Your analysis is very informative and I greatly appreciate this opportunity as it is not only an opportunity to assist others possibly but also to help further describe and inform my experiences using music.*”

## Data availability statement

The raw data supporting the conclusions of this article will be made available by the authors, without undue reservation.

## Ethics statement

The studies involving human participants were reviewed and approved by Bioethics Committee of the Institute of Neurobiology of the Universidad Nacional Autónoma de México. The patients/participants provided their written informed consent to participate in this study. Written informed consent was obtained from the participant/patient(s) for the publication of this case repor.

## Author contributions

EG-V developed the study concept and was contacted by the patient. RM performed the interviews. EG-V and RM analyzed and interpreted the interviews, drafted the manuscript, and approved the final version for submission.

## Conflict of interest

The authors declare that the research was conducted in the absence of any commercial or financial relationships that could be construed as a potential conflict of interest.

## Publisher’s note

All claims expressed in this article are solely those of the authors and do not necessarily represent those of their affiliated organizations, or those of the publisher, the editors and the reviewers. Any product that may be evaluated in this article, or claim that may be made by its manufacturer, is not guaranteed or endorsed by the publisher.
