# Supplementary material for: Case Report: “I got my brain back” A patient’s experience with music-induced analgesia for chronic pain
Source: Front Psychol. 2023 Apr 28;14:1141829. doi: 10.3389/fpsyg.2023.1141829 (PMC10175678; doi:10.3389/fpsyg.2023.1141829)
Supplement: Supplementary file 1 [file Data_Sheet_1.pdf]

Supplementary Material

"I got my brain back" A patient's experience with music induced analgesia for chronic pain

Roberto E. Mercadillo[1,2], Eduardo A. Garza-Villarreal[3*]

[1]Universidad Autónoma Metropolitana, Iztapalapa. Mexico City, Mexico

[2] CONACYT, Mexico City, Mexico

[3] Instituto de Neurobiología, Universidad Nacional Autónoma de México campus Juriquilla, Queretaro, Qro, Mexico

*** Correspondence:**
Eduardo A. Garza-Villarreal, MD, PhD
Assistant Professor,
Instituto de Neurobiología, Laboratorio B-03,
Universidad Nacional Autonoma de Mexico (UNAM) campus Juriquilla,
Boulevard Juriquilla 3001,
Santiago de Querétaro, Querétaro, México
C.P. 76230
Phone: (442) 238-1038
Email: egarza@comunidad.unam.mx

1 Phenomenological approach

Next, a narrative is shown following the neurophenomenological proposal that makes use of the experience communicated in first-person testimonials and emphasizes embodiment as a substrate of individuality (Varela et al., 1991; Díaz, 2022). Although it is shown in third person, the narrative tried to respect the explanatory style of the participant herself in order to understand, from her own perspective, what she considers most relevant from her experience. Some testimonials are shown verbatim to illustrate the narrative.

2 Accident, treatment and "cold turkey"

On New Year's Eve 2001, she was sitting in her car at a stop sign at the bottom of a hill; she was in a stick shift, so both legs were locked. Then a large car coming down the hill lost control and crashed into her and pushed her car onto a busy street.

At first it didn't hurt, but six hours later she had excruciating pain in her spine and couldn't walk and then she began to feel sharp like shards of blast sticking. She received two sets of facet joint injections. The first set "*worked wonderfully*" for eight months. But then the pain returned, and she asked for a second set: "*something went terribly wrong… The pain in my spine and hips increased. It was six*

months after the accident, I developed nerve pain on the top of my foot which felt like a hot poker – I don't' know if it was related to the Facet Joint Injections, but it happened shortly after the first set". When she stated that the second set of Facet Joint injections increased the pain in her lower spine and hips she was told "*it was a steroidal flare but no doctor can currently tell me what it was*". The pain increased almost permanently, and that's when she got her first prescription for oxycodone: "*I hated them because I was a single parent, I was working full-time, I was going to school, I was doing senior research, I was tired already. I didn't need something to make tired*"

Her pain was mostly in the lower spine and it felt like a type of crushed glass or like there was an ice pick in the middle between L3 and L4. She has a herniated bulging disc, nerve compression stenosis, and arthritis. Her second and third toes on her left foot have been tingling for 20 years since the accident and about five or six years after she developed sciatica: "*I was bending over doing some cleaning, and I just started to get this sharp pain down the back of my legs*". Medication didn't really help with that and it hurt so much that she tried to avoid a lot of activities.

She participated in a Comprehensive Chronic Pain program for the first time in 2019, when she was taking high doses of OxyContin. There they offered Multidisciplinary Rehabilitation, reiki, massages and acupuncture. But she found no relief may be because "[her*] central nervous system was so suppressed that they weren't effective at that time*". Last November, she did the 12-week program again and found incredible relief with reiki, "*like* [her] *whole body just vibrates…*"

She was angry about not finding alternatives to medication –"*I don't want that, I need my brain back*"– she thought. As of September 2021, she was taking nearly 300 milligrams of extended-release OxyContin and 300 milligrams of immediate-release oxycodone: "*I dropped 60 pounds in two months...it was killing me...not just the brain part, but physically so*". On Labor Day weekend that September she went cold turkey and that was the last time she took oxycodone. The first two weeks were horrible: "*as total heroin abstinence... I spent two weeks basically bedridden with all kinds of horrible biological stuff*".

In bed, while suffering from withdrawal symptoms and pain, she intuitively thought that she needed music through headphones. Her intuition may have come from her musical education; she is classically trained on the flute and piano, so she used to listen to Mozart or Beethoven anywhere, but she also used to listen to a wide range of music genres. At first, she would play music as loud as possible, and as the music played, she felt like it was repairing or feeding her brain. But she also noticed that some music was discordant or aggressive, or had too many sharps that made her feel like a cut or an ice pick right in her spine. So, by applying her musical knowledge, she began to fully focus on the harmony and the sound of notes, pitch, rhythm, and tones. Thus, she was able to identify that certain music provided her relief while another exacerbated her discomfort. She subscribed to a streaming service to access various types of music and select the one that will bring her relief. She is currently using music, reiki, acupuncture and Buddhist meditation for pain. But music has been more effective in many ways because: "*I have never felt better*".

3 Pain experience

Pain intensity and body location have fluctuated throughout these 20 years of treatment. When she is in pain, the experience is completely absorbing, she denotes not having thoughts or remembrances, either when she was taking medication or after stopped using it: "*I don't have memories that come when I'm in pain... the pain takes most on my focus and I cannot think of much else*".

4 Emotions and resting

She has experienced a variety of emotions throughout these 20 years, mostly negative: exhaustion, stress, sadness, anger, frustration and impatience. Each emotion has its own meaning. For example:

> "Exhaustion… like surrender to want to sleep forever... but just this whole exhaustion from the mental stress of pain, the physical stress of it".

> "Sadness… like it's something that I couldn't manage or do just because, having to deal with that one extra layer of problems in life".

> "Anger… about the medication for a long time… I was angry that there were no alternatives… I think those medications made me a bit narcissistic, because everything was all about me, and… my clouded fogged up little bubble that I lived in".

She has also felt happiness and relaxation with pain relief, either from medication or music. But even when happiness was present, other emotions persisted when using medication: stress, isolation, anger, and frustration with the pain. For example:

> "…all I wanted to do is just lay down and not move, and just zone out like watch television or something like that… just be a log and not interact with people because I was, first of all, always tired. I had no pleasure really in anything."

Sleep was a prominent issue since she had insomnia for a long time:

> "Sometimes I couldn't go to sleep because it just hurt too bad or would wake me up at night… if I slept more than four hours I'd wake up with that pain".

Now with music, sleep has improved:

> "I sleep like a rock… I usually wake up, I think I have trained myself to wake up in about five or five and a half hour... I'm working six hours, I hope to be able to do six and a half hours on a regular basis, so I think that's what I need about six and a half hours or seven hours... I usually feel pretty rested".

5 Cognition

Before the music, her sense of touch was dulled, whether she felt pain or relief: "*I was in a gray cloud and in a prison of flesh*". With the music "*not only* [her] *sense of touch came back but the gray cloud went away*".

She had difficulties concentrating while using narcotic medication. Using music "*has given* [her] *much more energy to pay attention to the things that matters…*"

In terms of learning and memory, she used to have a hard time remembering things and focusing on new material at work:

> "I found participating in meetings or trainings at work to be very difficult… I just didn´t have the patience... my brain felt like it was a brick of cheese the entire time".

With music she has increased her learning ability and is alert with an overdose of joy and interested in everything again:

> "I got my brain back... it's stimulated and awake, which has enhanced my ability to remember and learn".

Before music, she had a lot less patience and it carried over to how she communicated with other people. At work, she tended to be silent and limit interactions with her co-workers. In addition, she had difficulty holding a conversation. Now, with music: "*I am better able to communicate because I am no longer living in a gray cloud*".

Now she can run, bike and attend the gym regularly. The path to this has not been easy: "*It does take effort, you have to indulge yourself into the music, but what a pleasurable indulgence that is*!"

6 Music that relieves and causes pain

Now she probably uses music almost every waking hour, except when she is in a meeting. Every day she tries to find music that relieves the pain, so she is always exploring what kind of rhythms, tones and pitch are helping.

She has about 80 playlists and each playlist can have 15-100 tracks. From there she can make lists of music to relieve pain, but also to know what music causes her pain. She uses the Tidal streaming service because they have a large library of music. However, the 3D music most streaming services have sound like the band is playing in a gym with bad acoustics. High-fidelity headphones are crucial for good sound quality and the noise cancellation enhances the sound quality, so she can only hear the music. Although she has a stereo and albums, the stereo or the CD player isn't effective: "*headphones work better for the vibrations. It feels like the music is going right into your head*".

Some quiet songs motivate certain positions of her body. For example, when listening to Golden by Alexis Ffrench, she gets into a fetal position, that was a position she would take frequently when things were bad and "*it just came intuitive… it just gives [her] that weightless floating and it relieves pressure*".

For the song "Confines" by Black Pumas:

> "I like the lyrics... There's a particular part about if this light should burn out may we carry on gently down the street. But it's more the rhythm and the pitch and the tone, and just adding that string quartet just did so much for that song".
>
> "The guitar solo in Confines is too aggressive… the combination of the string quartet adds a lot. I've listened to the version without the string quartet and... it doesn't work for me at all, and the live version is terrible for me… so it's the quality of the sound and then the combination of the string quartet and Eric Burton's voice, the way, the tone and the pitch of his voice… His voice cracks on key and it's just amazing to me. I mean there's nothing phenomenal about his voice, there are much better singers out there but there's something about his voice… So, when I listen to this music I'm focused completely on the sound of the notes and the pitch and the rhythm and the tone… and that type of crescendo with strings or piano… I can visualize those notes like pulling the pressure in the pain."

Certain music has qualities that allow her to focus. For example, “Know you better” by Black Pumas is “*interesting because it is the ticking, it's the ticking of that snare. I meditate on that when the pain is really bad… the rhythm just kind of relaxes the body… this song is truly just more, so not using the notes it's using the mind and focusing on that ticking*”.

Not all music is for relaxation; relief can also be adding energy or vigor. For example, listen to The Panther by Manu Dibango to exercise: “*Not only I forget about the pain, I mean it doesn't resolve it but I can forget about... it can help me run faster and further because it's like getting a turbocharge. It's the best way I can explain, it's like an injection of energy*”.

Some music does not calm her down or makes her forget the pain, but instead focuses on pain to relief. For example, Schubert's Ave Maria with vocals by Renée Fleming makes her locate her pain and the sensation in her spine is very specific so she can feel the song along the vertebrate. That music makes her evoke beautiful memories or places, like cathedral ceilings. But she is surprised because:

> “I don't understand the language or how it even works being a religious song... I don't believe in the Christian God… I was raised strict Catholic, and as I got older I left that belief, let's just say my karma right over my dogma and… I belong to a group of existential Buddhist, I don't believe in the Christian God, I do believe Jesus was on the earth just like… Mohammed and all of the other prophets. But, I had really terrible experiences in the Catholic school. So I think it's the music itself because I wouldn't normally use it.”

She avoids some music because it reminds her of social situations evoking anger or stress. For example, The Poverty of Philosophy by Immortal Technique:

> “it's very political and these songs I love, but I can't listen to them because for these memories they cause a lot of stress… I don't go back to those memories, but if I would go back to them I would do it in a way that doesn't cause the stress that increases pain... the stress of the things that happened”.

Some music causes her pain because of the sound. For example, the Cello Suite No. 1 in G major by Yo-Yo Ma:

> “I hate to be a critic of Yo-Yo Ma… when listen to that song, especially on headphones, you can hear the scratching of the bow against the strings… it's scratchy, and it's loud, and it's almost equally loud as the notes registering in my ear, like someone putting nails down a chalkboard”.

Houses of Holy by Led Zeppelin is another example:

> “it's just as aggressive as any rock song... but it's the discordant sound if you listen to the guitar… they're kind of off right”.

7 References

Díaz, J.L. (2022). El enredo mente-cuerpo. Ciudad de México. Herder.

Varela, F., Thompson, E., and Rosch, E. (1991). The embodied mind: cognitive science and human experience. Cambridge: MIT Press.
